# Improvement of sleep quality after treatment in patients with lumbar spinal stenosis: a prospective comparative study between conservative versus surgical treatment

**DOI:** 10.1038/s41598-020-71145-0

**Published:** 2020-08-24

**Authors:** Jihye Kim, Seung Hun Lee, Tae-Hwan Kim

**Affiliations:** 1 Division of Infection, Department of Pediatrics, Kangdong Sacred Heart Hospital, Hallym University College of Medicine, Seoul, South Korea; 2 Department of Orthopedics, Spine Center, Hallym University Sacred Heart Hospital, Hallym University College of Medicine, 22, Gwanpyeong-ro, 170beon-gil, Dongan-gu, Anyang-si, Gyeonggi-do 14068 Republic of Korea

**Keywords:** Psychology, Diseases, Medical research, Circadian rhythms and sleep, Spine regulation and structure

## Abstract

Despite the importance of sleep and the evidence on its relationship with various chronic diseases, quality of sleep is not considered in patients with lumbar spinal stenosis (LSS). This prospective comparative study aimed to investigate the changes in sleep disturbance after treatment in patients with LSS. Patients with LSS and sleep disturbance (n = 201; 147 conservatively treated and 54 patients with surgical treatment) were included. The Pittsburgh sleep quality index (PSQI) was used to evaluate sleep quality. Propensity score matching was used to attenuate the potential bias. Clinical outcome of surgery, as determined by the Oswestry disability index, and the PSQI was compared between the two groups at 6 weeks, 3 months, and 6 months after enrollment. Multivariate logistic analysis was performed to adjust for possible confounders within the matched cohorts. Among the 201 patients, 96 (47.7%) patients were finally matched (48 patients in each group). Sleep quality was initially improved after treatment, regardless of the treatment method. Sleep quality in the surgical group was improved by 6 weeks after surgery and consistently improved during the 6-month follow-up period, despite less use of pain killer. Conversely, the improvement in sleep quality at 6-weeks following conservative treatment was not maintained during the follow-up, although the treatment outcome for LSS measured by ODI was continuously improved. After multivariate logistic regression analysis within propensity score matched cohorts, surgical treatment had a significantly greater chance to improve sleep quality compared to conservative treatment. The failure of sleep improvement in conservative group was significantly associated with depression presented by worse score in Hamilton depression rating scale, and more severe degree of foraminal-type stenosis, which should be carefully considered for conservative treatment of LSS patients with sleep disturbance.

## Introduction

Lumbar spinal stenosis (LSS) refers to any type of narrowing of the lumbar spinal canal including central spinal canal or intervertebral foramina^1^, and it is one of the most common pathologic conditions affecting the spine^2^. The prevalence of degenerative LSS is reported ranging from 1.7 to 13.1%^3–5^, and it sharply increases with age to 47.2% in the 60 s^2^. Pain is main cause for seeking treatment in patients with LSS, and most commonly involves lower back, buttock, thigh and leg. Patients with LSS typically presents neurogenic claudication, which shows pain appearing with standing or lumbar extension, aggravated by walking, and relieved by sitting or forward flexion^6,7^. Most patients are treated conservatively with pain killers, injection physiotherapy, and rehabilitation. However, for patients whose symptom do not improve after conservative treatment, surgical treatment which includes decompression of spinal canal with or without spinal fusion can be performed to improve their clinical symptoms^6^.

Sleep is essential for restoration of physiologic function in addition to learning, memory, and cognition^8,9^. Disturbed sleep not only negatively affects quality of life but also causes mental^10^ and physical^11^ illness such as cardiovascular diseases, diabetes, and cancer, and eventually increases mortality^12^. Especially in older populations with chronic medical conditions, quality of sleep is closely linked to their life expectancy as well as quality of life^12–18^. However, despite the essential role of sleep in mental and physical health of older populations, quality of sleep is not considered in patients with LSS. The Oswestry disability index, the most commonly used disability index for LSS, simply evaluates the presence of pain-related sleep disturbance^19^. The short-form 36-item questionnaire (SF-36) and the EuroQol (EQ-5D), the most widely used health-related quality of life measures for patients with LSS, do not measure the quality of sleep at all^20^.

Cross-sectional studies investigated the quality of sleep in other musculoskeletal diseases such as osteoarthritis of the knee, and rotator cuff tear^21,22^, and recent prospective case-series measured their changes after surgical treatment^23,24^. In such joint diseases, pain is generally reduced during sleep, because the gravitational joint force is decreased in the lying position. However, in clinical practice, we frequently observe that symptoms of patients with LSS are aggravated having difficulty in falling asleep. Patients with LSS have a relatively smaller spinal canal during lumbar extension compared to lumbar flexion^25^. During sleep, patients with LSS remain in a prolonged lying position with lumbar extension, which causes significant narrowing of the spinal canal and aggravates symptoms from LSS^26^. Therefore, the pattern of sleep disturbance in patients with LSS and its changes after treatment is thought to be significantly different from other joint diseases.

Despite the importance of sleep and the distinct positional intolerance for lying during sleeping in LSS patients, there have been little studies that investigated sleep disturbance in LSS patients. We therefore planned a prospective comparative study to compare the changes in sleep disturbance in patients with LSS who either received conservative or surgical treatment.

## Materials and methods

### Study patients

**Figure 1:**
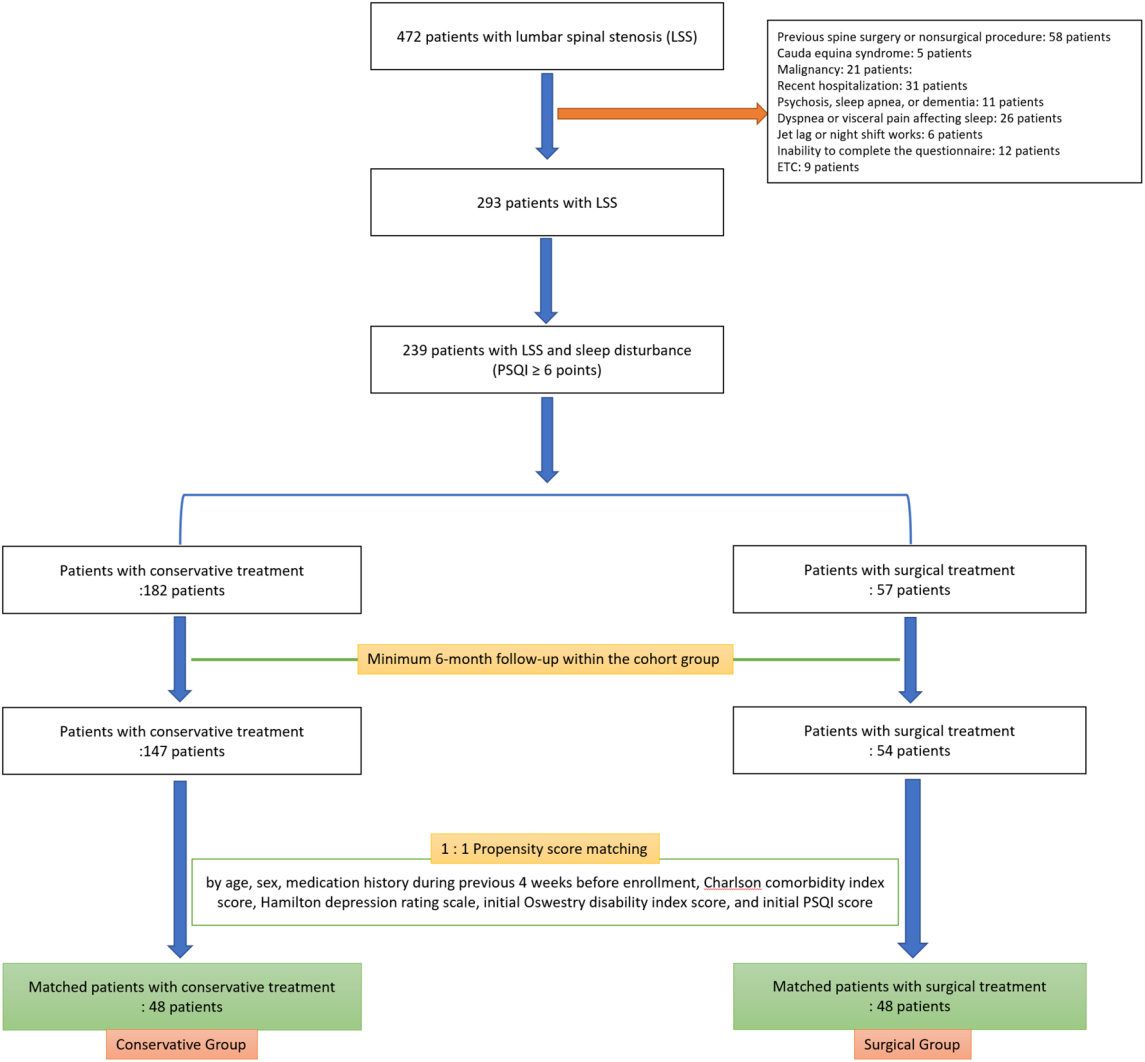
Patient enrollment.

A prospective cohort study was performed on patients with LSS who visited our institution’s spine center between May 2019 and October 2019. We excluded the following: patients who had either received surgical treatment for LSS, or a nonsurgical spinal procedure that included pain block within 6 weeks before the enrollment; cauda equina syndrome; malignancy; recent hospitalization; diagnosis of psychosis, sleep apnea, or dementia; dyspnea or visceral pain affecting sleep within 4 weeks before enrollment; recent overseas travel with jet lag and night shift works, and finally the inability to complete the questionnaire (Fig. 1).

### Measurement of sleep disturbance: Pittsburgh sleep quality index (PSQI)

Sleep disturbance was evaluated with the standardized, local language version of the Pittsburgh sleep quality index (PSQI), which evaluates sleep habits during the last month^27–29^. This self-report questionnaire consists of 19 questions with seven subcategories: sleep quality, latency, duration, and disturbance; habitual sleep efficiency; use of sleep medications, and daytime dysfunction. The sum of the scores from all seven subcategories produces a global score (the PSQI score) ranging from 0 to 21 with higher scores associated with a poorer quality of sleep. A PSQI score over 5 points showed a diagnostic sensitivity of 89.6% and specificity of 86.5% (kappa = 0.75, p < 0.001) in distinguishing good and poor sleepers^27^, and PSQI of ≥ 6 points is generally accepted as indicating sleep disturbance^30^.

### Covariates

Demographic data and medical history included age, body mass index (BMI), education, precise medication history during the previous 4 weeks before enrollment, and medical comorbidities evaluated by the Charlson comorbidity index. Information on lifestyle associated with sleep included occupation (none, sedentary or physical worker), smoking (within 2 h before bedtime), alcohol (within 2 h before bedtime), and caffeine intake (within 6 h before bedtime). The presence of chronic joint pain in the upper or lower extremities was included. Other questionnaires included the Oswestry disability index (ODI) score, and the Hamilton depression rating scale.

The following predefined radiologic parameters on lumbar stenosis severity were measured with MRI. The dural sac cross-sectional area (mm^2^) was measured at the most severe level of stenosis. At this level, we evaluated the morphologic grade of central-type stenosis based on the morphology of the dural sac^31^ and the morphologic grade of foraminal-type stenosis^32^.

### Grouping and propensity score matching

Patients with LSS who had sleep disturbance (PSQI ≥ 6 points) were enrolled for the longitudinal study. The cohort of patients was divided into two groups according to the method of treatment: conservative group and surgical group (Fig. 1). Propensity score matching was used to attenuate potential selection bias between the two groups. Sleep quality at the time of enrollment could be influenced by the method of treatment of LSS during the previous 4 weeks. Therefore, we excluded patients who had received any surgical or nonsurgical procedures within the previous 6 weeks, and propensity score matching was done for the precise medication history, including the use of pregabalin, gabapentin, and opioids during the previous 4 weeks. The propensity score model additionally consisted of the covariates including age, sex, Charlson comorbidity index score, Hamilton depression rating scale, initial ODI score, and initial global PSQI score. Patient characteristics and infection profiles were compared between the two groups before and after the propensity score matching (Tables 1, 2).

**Table 1:** Comparison of initial patients’ characteristics.

	Unmatched cohort			Matched cohort		
	Conservative group	Surgical group	p-value	Conservative group	Surgical group	p-value
Number of patients	147	54		48	48	
Age	69.6 ± 10.5	71.9 ± 6.7	0.067	71.4 ± 9.3	71.7 ± 7.0	0.873
Sex (male:female)	61:86 (1:1.4)	17:37 (1:2.2)	0.197	17:31 (1:1.8)	16:32 (1:2.0)	0.830
BMI (kg/m^2^)	24.4 ± 3.1	24.8 ± 3.3	0.400	24.1 ± 3.2	24.9 ± 3.4	0.248
Charlson comorbidity index score	1.4 ± 1.3	1.3 ± 1.2	0.481	1.3 ± 1.3	1.3 ± 1.2	0.833
Hamilton depression rating scale	12.7 ± 8.8	13.7 ± 8.4	0.480	13.8 ± 8.4	13.5 ± 8.4	0.885
Oswestry disability index score	38.5 ± 17.7	41.9 ± 18.4	0.234	40.1 ± 16.5	41.0 ± 17.7	0.810
**Visual analogue scale pain**						
Back pain	4.9 ± 1.3	6.1 ± 1.7	< 0.001	5.5 ± 1.5	6.0 ± 1.8	0.120
Leg pain	5.1 ± 1.6	6.8 ± 1.8	< 0.001	6.2 ± 1.3	6.7 ± 1.9	0.094
**Education**			0.621			0.797
Less than high school	86 (59)	28 (52)		27 (56)	25 (52)	
High school	39 (27)	18 (33)		13 (27)	16 (33)	
College and above	22 (15)	8 (15)		8 (17)	7 (15)	
**Occupation**			0.784			0.603
None	112 (76)	43 (80)		40 (83)	38 (79)	
Sedentary	25 (17)	7 (13)		4 (8)	7 (15)	
Physical worker	10 (7)	4 (7)		4 (8)	3 (6)	
**Medication during the previous 4 weeks**						
Pregabalin or Gabapentin	84 (57)	39 (72)	0.052	27 (56)	34 (71)	0.138
Opioids	65 (44)	34 (63)	0.018	24 (50)	29 (60)	0.305
Smoking within 2 h of bedtime	24 (16)	7 (13)	0.558	8 (17)	7 (15)	0.779
Alcohol within 2 h of bedtime	19 (13)	6 (11)	0.730	5 (10)	6 (12)	0.749
**Caffeine within 6 h of bedtime**						
Coffee	15 (10)	4 (7)	0.786	4 (8)	4 (8)	1.000
Tea	20 (14)	6 (11)	0.640	8 (17)	4 (8)	0.217
**Chronic joint pain**						
Lower extremities	35 (24)	12 (22)	0.814	12 (25)	11 (23)	0.811
Upper extremities	22 (15)	9 (17)	0.767	8 (17)	9 (19)	0.789
**Pittsburgh sleep quality index**						
Global score	12.1 ± 3.3	12.6 ± 3.6	0.365	12.5 ± 3.1	12.3 ± 3.2	0.748
Sleep quality	1.9 ± 0.9	1.9 ± 1.1	0.836	2.1 ± 0.8	1.9 ± 1.1	0.340
Sleep latency	3.2 ± 2.1	3.9 ± 2.2	0.027	3.3 ± 2.1	4.0 ± 2.1	0.111
Sleep duration	2.1 ± 1.0	2.1 ± 1.0	0.809	2.2 ± 1.0	2.0 ± 1.0	0.543
Sleep efficiency	1.4 ± 0.7	1.2 ± 0.6	0.019	1.5 ± 0.7	1.1 ± 0.5	0.001
Sleep disturbance	1.7 ± 0.6	1.7 ± 0.7	0.883	1.8 ± 0.6	1.7 ± 0.7	0.442
Use of sleep medication	0.4 ± 0.7	0.4 ± 0.7	0.702	0.3 ± 0.6	0.3 ± 0.7	0.874
Daytime dysfunction	1.3 ± 0.9	1.4 ± 1.0	0.655	1.4 ± 1.0	1.4 ± 1.0	0.833

Data were presented by number (%) of patients or mean ± standard deviation.

**Table 2:** Comparison of initial radiologic profile.

	Unmatched cohort			Matched cohort		
	Conservative group	Surgical group	p-value	Conservative group	Surgical group	p-value
Dural sac cross-sectional area (mm^2^) at the most severe level	57 ± 34	45 ± 30	0.014	51 ± 29	45 ± 32	0.323
Morphologic grade of stenosis at the most severe level						
**Central-type stenosis by Schizas et al.**						
A or B	37 (25)	8 (15)	0.147	10 (21)	7 (15)	0.530
C	66 (45)	23 (43)		21 (44)	19 (40)	
D	44 (30)	23 (43)		17 (35)	22 (46)	
**Foraminal-type stenosis by Lee et al.**						
Grade 0 or 1	28 (19)	7 (13)	0.300	10 (21)	7 (15)	0.250
Grade 2	60 (41)	19 (35)		22 (46)	17 (35)	
Grade 3	59 (40)	28 (52)		16 (33)	24 (50)	

Data were presented by number (%) of patients or mean ± standard deviation.

### Treatment protocol and follow-up measures

The conservative treatment protocol included a nonsteroidal anti-inflammatory agent, physical therapy, education for exercising at home, and pregabalin, gabapentin or opioids if tolerated. Patients were guided to take the provided oral medications with strict adherence for the first 4 weeks and freely thereafter on the basis of their need. Surgical treatment included posterior decompression with or without spinal fusion under general anesthesia. Patients received postoperative intravenous patient-controlled analgesia for 24 h. Additionally, patients received oxycodone extended-release 5–10 mg by mouth during the 2-week postoperative period. Follow-up measurement of ODI scores and PSQI scores was performed at 6 weeks, 3 months, and 6 months after enrollment, together with a detailed history of prescribed medication.

### Statistical analysis

Sample size was estimated from the result of our preliminary cohort study which included 20 patients with sleep disturbance. After 6-month follow-up, mean PSQI global score was 6.2 points for surgical group (standard deviation = 2.4) and 7.7 points for conservative group. Considering a power of 0.90 (alpha = 0.05), we calculated a minimum sample size of 27 patients.

The effect of surgical treatment on the improvement of the PSQI score at the 6-month follow-up was evaluated using multivariate logistic regression analysis to adjust for confounding factors. Multivariate adjustment was initially done for the variables included in the propensity score matching (model 1) and also after adding radiologic variables (model 2). According to previous reports, a change in global PSQI score ≥ over three points or more was regarded as a minimal clinically important difference^1^.

The statistical tests were two-tailed, and a p-value of < 0.05 indicated significance. Analyses were performed using SPSS 24 (SPSS Inc., Chicago, Illinois, USA).

### Ethics

This study was designed and conducted using the STROBE format (Strengthening the Reporting of Observational studies in Epidemiology) guidelines^33^. This study was approved by the institutional review board of Hallym University Sacred Heart Hospital (2019-04-002), and written informed consent was obtained from all patients.

## Results

### Enrollment and grouping

A total of 239 patients with LSS and sleep disturbance (PSQI ≥ 6 points) were identified for our study (Fig. 1). A 6-month follow-up was only available for 201 patients (84.1%), consisting of 147 patients with conservative treatment and 54 patients with surgical treatment. Using the propensity score, 96 (47.7%) of the 201 patients were finally matched (48 patients in each group; Fig. 1). Among 48 patients of the surgical group, 9 patients underwent only decompressive surgery, and 39 patients underwent additional spinal fusion.

### Comparison of initial patients’ characteristics

Before matching, the surgical group used opioids more frequently during the 4 weeks before the enrollment than the conservative group (63% versus 44%, p = 0.018, Table 1). However, this difference was not significantly different after matching (60% versus 50%, p = 0.305, Table 1). The other variables of propensity score matching which include age, sex ratio, Charlson comorbidity index score, Hamilton depression rating scale, initial ODI score, initial global PSQI score, and use of pregabalin or gabapentin during the previous 4 weeks were more balanced after matching (Table 1).

### Comparison of initial radiologic profile

Before matching, the surgical group had a statistically smaller dural sac cross-sectional area (45 mm^2^ versus 57 mm^2^, p = 0.014, Table 2). However, it was not significantly different after matching (45 mm^2^ versus 51 mm^2^, p = 0.323, Table 2), even though matching was not done for the dural sac cross-sectional area. Morphological grades of stenosis at the most severe level, presented by either central-type stenosis or foraminal-type stenosis, were not significantly different between the two groups before and after matching (Table 2).

### Comparison of prescribed medications within the matched cohorts

There were no significant differences in prescribed medication use during the previous 4 weeks including NSAIDs, opioids, pregabalin or gabapentin, and sleep medication (Table 3). However, 6 weeks after enrollment, opioid, and pregabalin or gabapentin were less frequently prescribed in the surgical group. At 3 and 6-months after enrollment, NSAIDs, opioids, and pregabalin or gabapentin were also less frequently prescribed in the surgical group. There were no significant differences in the use of sleep medication during the follow-up period between the two groups.

**Table 3:** Comparison of prescribed medications during the follow-up period.

	Conservative group	Surgical group	p-value
**NSAIDs**			
During previous 4 weeks	46 (96)	44 (92)	0.399
At 6-week follow-up	45 (94)	40 (83)	0.109
At 3-month follow-up	42 (88)	7 (15)	< 0.001
At 6-month follow-up	44 (92)	2 (4)	< 0.001
**Opioids**			
During previous 4 weeks	24 (50)	29 (60)	0.305
At 6-week follow-up	34 (71)	23 (48)	0.022
At 3-month follow-up	27 (56)	3 (6)	< 0.001
At 6-month follow-up	19 (38)	1 (2)	< 0.001
**Pregabalin or gabapentin**			
During previous 4 weeks	27 (56)	34 (71)	0.138
At 6-week follow-up	28 (58)	15 (31)	0.008
At 3-month follow-up	26 (54)	4 (8)	< 0.001
At 6-month follow-up	24 (50)	2 (4)	< 0.001
**Sleep medication**			
During previous 4 weeks	12 (25)	11 (23)	0.811
Type of sleep medication			0.307
Hypnotics	8	5	
Benzodiazepine	3	4	
Anti-depressant	1	2	
At 6-week follow-up	12 (25)	11 (23)	0.811
At 3-month follow-up	13 (27)	11 (23)	0.637
At 6-month follow-up	14 (29)	10 (21)	0.346

Data were presented by number (%) of patients or mean ± standard deviation.

### Comparison of changes in clinical profile within the matched cohorts

**Figure 2:**
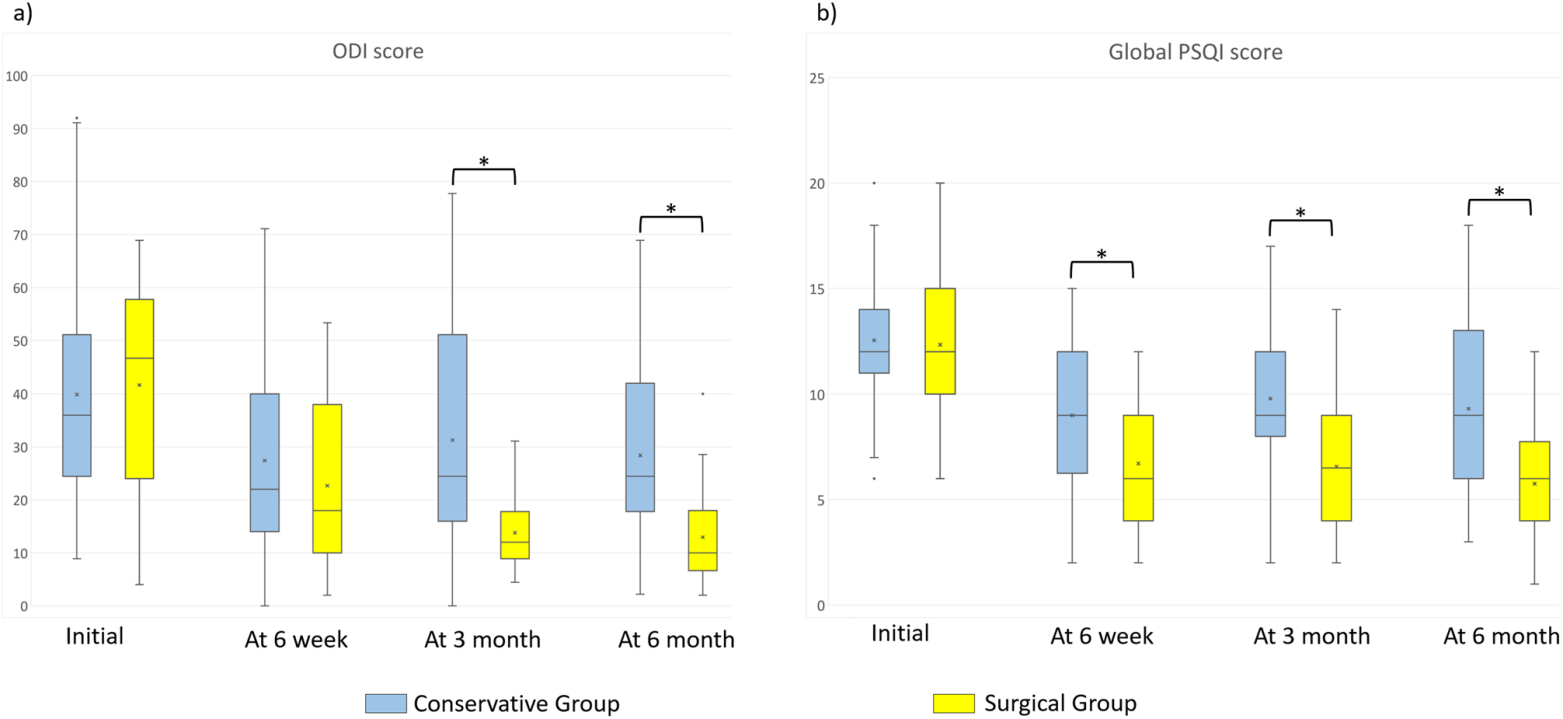
Comparison of changes in clinical profile within the matched cohorts (*significant intergroup difference of p < 0.05). (**a**) Comparison of Oswestry Disability Index (ODI) score. (**b**) Comparison of Pittsburgh sleep quality index (PSQI) score. Middle line in the box: median value; x mark within the box: mean value; upper and lower edges of box: interquartile range including 50% of the observations; dots outside box: outliers. Whisker lines extend for 1.5 times the interquartile range.

There were no differences in the initial ODI score and the initial global PSQI score between the two groups. However, the surgical group had significantly lower ODI scores at 3 and 6-months follow-up, and significantly lower global PSQI scores throughout the follow-up (Table 4, Fig. 2).

**Table 4:** Comparison of changes in clinical profile within the matched cohorts.

	Conservative group	Surgical group	p-value
**Oswestry disability index score**			
Initial	40.1 ± 16.5	41.0 ± 17.7	0.810
At 6-week follow-up	25.9 ± 15.3	25.7 ± 14.4	0.949
At 3-month follow-up	23.4 ± 13.5	13.4 ± 6.4	< 0.001
At 6-month follow-up	19.1 ± 12.8	12.1 ± 7.9	0.002
**PSQI global score**			
Global score			
Initial	12.5 ± 3.1	12.3 ± 3.2	0.748
At 6-week follow-up	9.0 ± 3.2	6.7 ± 2.9	< 0.001
At 3-month follow-up	9.8 ± 3.1	6.6 ± 2.9	< 0.001
At 6-month follow-up	9.3 ± 4.0	5.8 ± 2.6	< 0.001
**Decrease of global score from initial value ≥ 3 (= improvement of sleep quality)**			
Initial	–	–	
At 6-week follow-up	29 (60)	37 (77)	0.078
At 3-month follow-up	22 (46)	39 (81)	< 0.001
At 6-month follow-up	24 (50)	41 (85)	< 0.001
**Scores of 7 subcategories in PSQI**			
Sleep quality			
Initial	2.1 ± 0.8	1.9 ± 1.1	0.340
At 6-week follow-up	0.9 ± 0.9	0.7 ± 0.5	0.348
At 3-month follow-up	1.1 ± 0.8	0.9 ± 0.7	0.111
At 6-month follow-up	1.0 ± 1.0	0.6 ± 0.6	0.021
Sleep latency (minutes)			
Initial	3.3 ± 2.1 (46 ± 25)	4.0 ± 2.1 (52 ± 26)	0.032
At 6-week follow-up	2.6 ± 2.0 (38 ± 19)	1.8 ± 1.7 (30 ± 16)	0.111
At 3-month follow-up	2.9 ± 2.2 (40 ± 22)	1.4 ± 1.0 (25 ± 17)	0.001
At 6-month follow-up	2.6 ± 2.0 (37 ± 23)	1.3 ± 1.5 (24 ± 15)	0.001
Sleep duration (minutes)			
Initial	2.2 ± 1.0 (323 ± 63)	2.0 ± 1.0 (326 ± 61)	0.543
At 6-week follow-up	1.9 ± 1.0 (343 ± 63)	1.6 ± 1.0 (364 ± 60)	0.230
At 3-month follow-up	1.8 ± 1.0 (338 ± 72)	1.4 ± 1.0 (375 ± 68)	0.059
At 6-month follow-up	1.7 ± 1.1 (343 ± 78)	1.1 ± 1.0 (373 ± 70)	0.152
Sleep efficiency			
Initial	1.5 ± 0.7	1.1 ± 0.5	0.001
At 6-week follow-up	1.2 ± 0.9	0.9 ± 1.0	0.146
At 3-month follow-up	1.4 ± 1.0	1.0 ± 0.9	0.029
At 6-month follow-up	1.4 ± 1.1	1.1 ± 1.0	0.184
Sleep disturbance			
Initial	1.8 ± 0.6	1.7 ± 0.7	0.442
At 6-week follow-up	1.1 ± 0.8	0.7 ± 0.6	0.011
At 3-month follow-up	1.1 ± 0.8	0.8 ± 0.6	0.048
At 6-month follow-up	1.1 ± 1.0	0.5 ± 0.7	< 0.001
Use of sleep medication			
Initial	0.3 ± 0.6	0.3 ± 0.7	0.874
At 6-week follow-up	0.3 ± 0.6	0.2 ± 0.4	0.345
At 3-month follow-up	0.3 ± 0.6	0.3 ± 0.5	0.454
At 6-month follow-up	0.4 ± 0.6	0.2 ± 0.5	0.260
Daytime dysfunction			
Initial	1.4 ± 1.0	1.4 ± 1.0	0.833
At 6-week follow-up	1.1 ± 0.8	0.8 ± 0.7	0.051
At 3-month follow-up	1.0 ± 0.8	0.7 ± 0.8	0.030
At 6-month follow-up	1.2 ± 0.9	0.7 ± 0.7	0.012

Data were presented by number (%) of patients or mean ± standard deviation.

**Figure 3:**
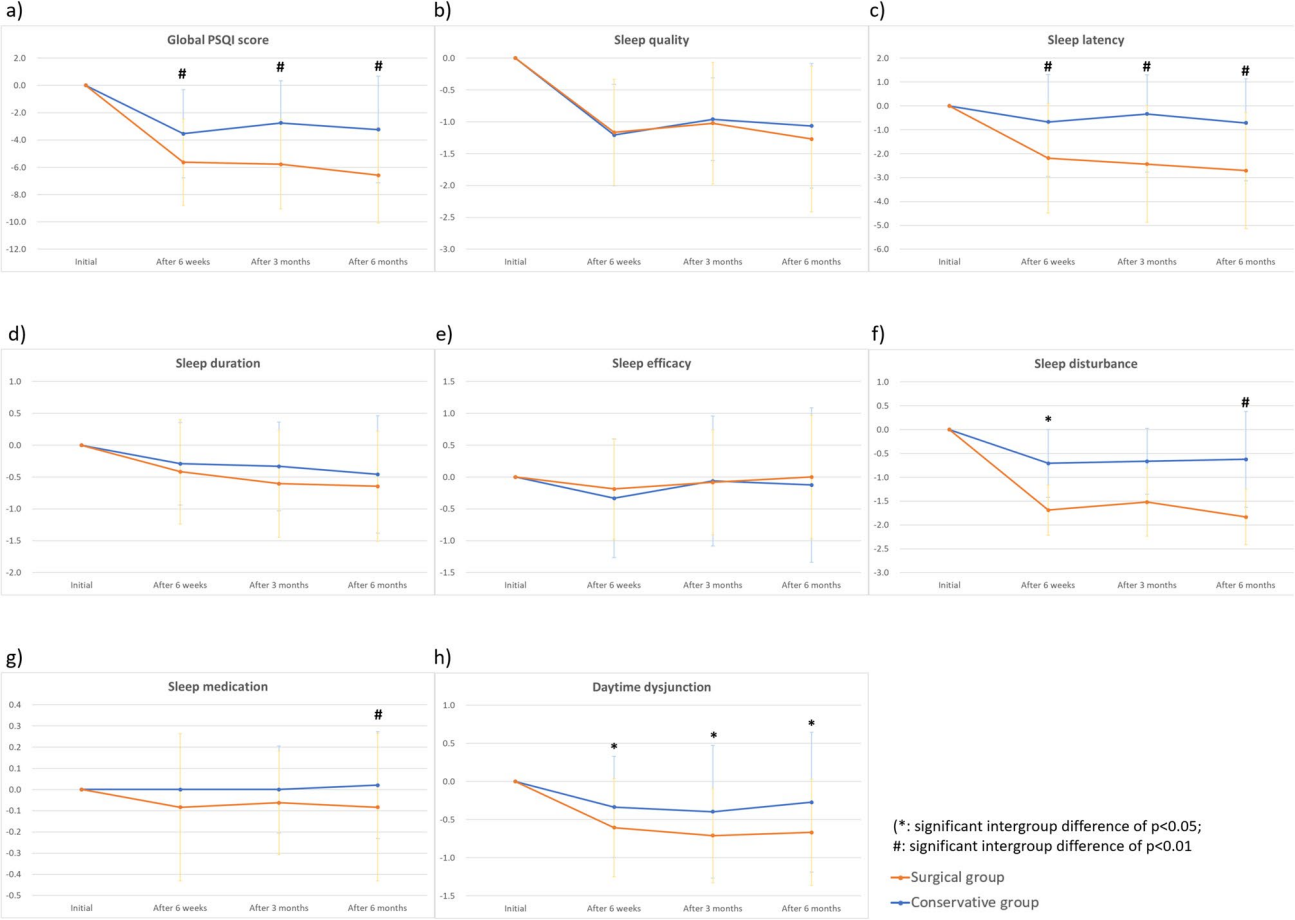
Changes in global PSQI score and scores of its seven subcategories between the three time points (at 6 weeks, 3 months, and 6 months) and their initial values. (**a**) Global Pittsburgh sleep quality index (PSQI) score. (**b**) Sleep quality index. (**c**) Sleep latency index. (**d**) Sleep duration index. (**e**) Sleep efficacy index. (**f**) Sleep disturbance index. (**g**) Sleep medication index. (**h**) Daytime dysfunction index. (*significant intergroup difference of p < 0.05; ^#^significant intergroup difference of p < 0.01).

**Figure 4:**
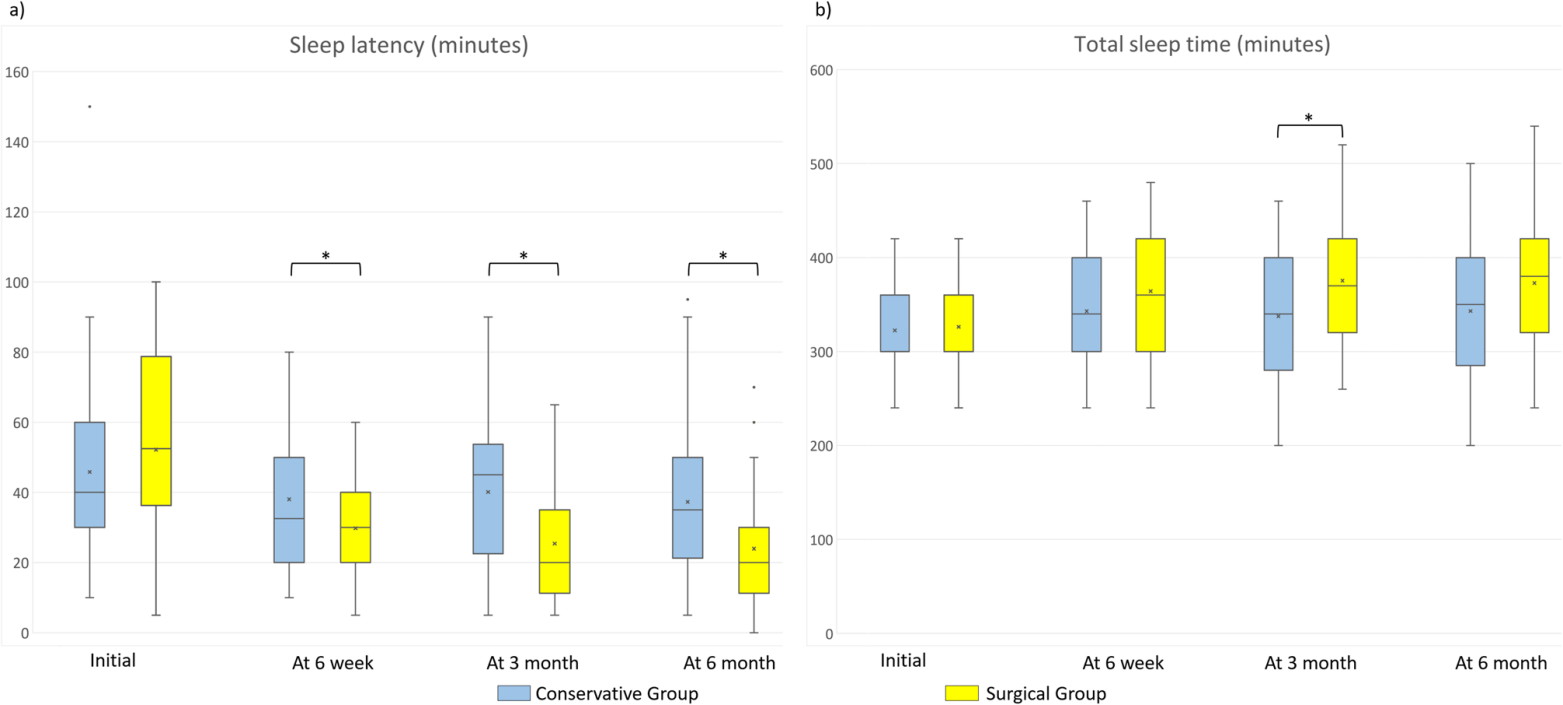
Comparison of sleep latency and total sleep time assessed by actual minutes (*significant intergroup difference of p < 0.05). (**a**) Comparison of sleep latency assessed by minutes. (**b**) Comparison of total sleep time assessed by minutes.

Changes of the global PSQI score and the scores of its seven subcategories from the initial values were compared between the two groups (Fig. 3). As the treatment continued, there were decreasing trends in these values regardless of the treatment group, which means clinically improved sleep. However, the surgical group had lower (clinically improved) values in the global PSQI score (Fig. 3a), sleep latency index (Fig. 3c), sleep disturbance index (Fig. 3f), and daytime dysfunction index (Fig. 3h) than the conservative group at both 6-week and 6-month follow-up. Despite the significant difference in sleep latency (minutes), there was no difference in total sleep time (minutes) between the two groups at 6-months after enrollment (Fig. 4).

### Effect of surgical treatment on the improvement of PSQI score at 6-month follow-up: multivariate analysis

At the 6-month follow-up, clinically meaningful improvement in sleep (a decreased global PSQI score ≥ 3 points) was observed in 50% (24 of 48 patients) of the conservative group, and 85.4% (41 of 48 patients) of the surgical group (Table 5). The multivariate logistic regression analysis shows that the surgical group had a significantly greater chance of improved sleep than the conservative group by the 6-month follow-up, in both matched and whole cohort.

**Table 5:** Effect of surgical treatment on the improvement of PSQI score at 6-month follow-up: multivariate analysis.

Cohort	Model	Group	Odds ratios	95% confidence interval	p-value
Matched cohort	Model 1	Conservative group	–	–	–
		Surgical group	8.589	(2.755, 26.772)	< 0.001
	Model 2	Conservative group	–	–	–
		Surgical group	8.810	(2.705, 28.690)	< 0.001
Whole cohort	Model 1	Conservative group	–	–	–
		Surgical group	8.802	(3.505, 22.104)	< 0.001
	Model 2	Conservative group	–	–	–
		Surgical group	8.264	(3.267, 20.907)	< 0.001

Model 1: adjusted for age, sex, Charlson comorbidity index score, Hamilton depression rating scale, initial Oswestry disability index score, and initial global PSQI score.

Model 2: adjusted for age, sex, Charlson comorbidity index score, Hamilton depression rating scale, initial Oswestry disability index score, initial global PSQI score, dural sac cross-sectional area, central-type stenosis by Shizas et al., and foraminal-type stenosis by Lee et al.

Data were presented by number (%) of patients.

### Factors associated with failure in sleep improvement: subgroup analysis within the whole conservative cohort

Although significant sleep improvement occurred in 85.2% (46 of 54 patients) of surgical group, sleep improvement occurred only in 45.6% (67 of 147 patients) of conservative group. Factors possibly associated poor outcome after conservative treatment were investigated comparing independent variables between the patients with and without sleep improvement within the whole conservative subgroup (Table 6). After subgroup analysis, patients without significant sleep improvement after conservative treatment showed significantly worse score in Hamilton depression rating scale (p = 0.022), and more severe degree of foraminal-type stenosis (p = 0.026) than patients with sleep improvement (Table 6).

**Table 6:** Factors associated with failure in sleep improvement: subgroup analysis within the whole conservative cohort.

	Whole conservative group		
	Increase in PSQI ≥ 3	Increase in PSQI < 3	p-value
Number of patients	67	80	
Age	70.2 ± 10.1	69.0 ± 10.8	0.948
Sex (male:female)	28:39 (1:1.4)	33:47 (1:1.4)	0.947
BMI (kg/m^2^)	24.0 ± 3.1	24.8 ± 3.1	0.123
Charlson comorbidity index score	1.6 ± 1.4	1.2 ± 1.3	0.095
Hamilton depression rating scale	11.0 ± 7.6	14.2 ± 9.2	0.022
Oswestry disability index score	36.4 ± 17.8	40.2 ± 17.5	0.197
**Visual analogue scale pain**			
Back pain	4.8 ± 1.4	4.9 ± 1.3	0.903
Leg pain	4.9 ± 1.6	5.3 ± 1.6	0.105
Dural sac cross-sectional area (mm^2^) at the most severe level	53 ± 30	60 ± 36	0.179
Morphologic grade of stenosis at the most severe level			
**Central-type stenosis by Schizas et al**			
A or B	16 (24)	21 (26)	0.779
C	29 (43)	37 (46)	
D	22 (33)	22 (28)	
**Foraminal-type stenosis by Lee et al**			
Grade 0 or 1	12 (18)	16 (20)	0.026
Grade 2	35 (52)	25 (31)	
Grade 3	20 (30)	39 (49)	

Data were presented by number (%) of patients or mean ± standard deviation.

## Discussion

To the best of our knowledge, this is the first study to compare the effect of treatment for LSS on sleep quality, between patients managed conservatively and those treated surgically. Our findings suggest that sleep quality, as measured by the PSQI, was improved after treatment, regardless of the treatment method (Table 3, Fig. 3). However, the surgical group showed more rapid improvement of sleep after treatment. Sleep latency index, sleep disturbance index and index of daytime dysfunction were significantly lower in the surgical than in the conservative group, even 6 weeks after treatment (Fig. 3). In addition, the surgical group showed a consistent improvement in sleep after treatment. At 6 weeks, a clinically significant improvement in sleep (a decrease global PSQI score ≥ 3 points) was shown in both the conservative and the surgical group, but there was no difference between them. However, by the 6-month follow-up, clinical improvement of sleep was shown in 46% (22 of 48 patients) in the conservative group, and 85% (41 of 48 patients) in the surgical group and the difference was significant (p < 0.001, Table 4).

Rapid improvement in sleep seen as early as 6 weeks after surgical treatment for LSS is noteworthy. According to the studies that examined changes in sleep after surgical treatment for osteoarthritis of the knee and rotator cuff tear, sleep worsened 6 weeks after surgery, and significant improvement was only achieved 3 months after surgery^23,24^. However, in our cohort, global PSQI scores decreased as early as 6 weeks after surgical treatment (12.3–6.7, Table 4). We believe that immediate decompression or widening of the lumbar spinal canal by surgery enables such improvement in sleep. In patients with knee osteoarthritis or rotator cuff disease, gravitational joint force is minimized in a supine lying position for sleep. Therefore, their joint pain generally decreases during sleeping, and postoperative pain improvement during sleeping might not be notable early after surgery. Conversely, in LSS patients, their narrow intervertebral foramen and central spinal canal further decreases during lumbar extension compared to that during lumbar flexion^25^. During sleeping, LSS patients inevitably remain in a prolonged lying position with lumbar extension, causing significant narrowing of the spinal canal and intensifying LSS symptoms. A recent published study demonstrated that foraminal stenosis, which causes leg pain, is independently associated with sleep disturbance in LSS patients^26^. Surgical decompression of the spinal canal in LSS patients can immediately and permanently enlarge foraminal size, more effectively improve leg pain and resultantly improve sleep quality. In clinical practice we frequently observe LSS patients who needed to assume an uncomfortable lateral decubitus position with lumbar flexion even after pain block, being able to adopt a comfortable supine position with lumbar extension immediately after surgery. As expected, subgroup analysis of conservative cohort identified that patients without significant sleep improvement after conservative treatment showed more severe degree of foraminal-type stenosis (p = 0.026) than patients with sleep improvement (Table 7).

**Table 7:** Correlation between changes in pain and disability scale and changes in global PSQI score within the whole cohort.

	All		Conservative group		Surgical group	
	Correlation coefficient	p-value	Correlation coefficient	p-value	Correlation coefficient	p-value
**Changes in visual analogue scale**						
Back pain	0.360	< 0.001	0.171	0.038	0.343	0.011
Leg pain	0.555	< 0.001	0.297	< 0.001	0.681	< 0.001
Changes in Oswestry disability index score	0.175	0.013	0.223	0.007	0.207	0.133

Another characteristic finding of sleep improvement in the surgical group compared to that in the conservative treatment is its consistent effect on sleep quality. In the surgical group, the ODI and the global PSQI scores were consistently decreased during the follow-up period. Although the use of pain killers was significantly lower in the surgical group (Table 3), the percentage of patients who showed clinically meaningful improvement in the global PSQI score increased from 77 to 85% (Table 4). However, in the conservative group, the ODI score was also consistently decreased during the 6-month follow-up (Table 4), but the percentage of patients who showed clinically meaningful improvement in the global PSQI score rather decreased from 60 to 50% during the same period (Table 4).

The major limitation of our study is that we could not eliminate the risk of bias resulting from unknown confounders because of the non-randomized study design. To reduce such bias, we tried to investigate all possible variables which can affect sleep, including the degree of depression, education level, occupation, presence of degenerative joint disease known to be related to the sleep disturbance, extent of lumbar stenosis, and lifestyle factors such as smoking, alcohol intake, and caffeine intake. Then, we matched such variables between the conservative and the surgical group using propensity score matching (Tables 1, 2). Finally, we performed a multivariate analysis to additionally adjust such possible confounders within the matched cohorts.

Our findings must be carefully interpreted. Several patients in the conservative group were using intermittent pain killers including NSAIDS, opioid and pregabalin or gabapentin for LSS at the time of their enrollment (Table 3). However, 60% of patients (29 of 48 patients, Table 4) exhibited significant improvement in sleep within 6 weeks of enrollment in the conservative group. Such improvement might be attributable to the additional physical therapy or education program provided as part of our treatment. However, possible influence of unknown confounders should not be disregarded.

Our findings indicate that surgical treatment for LSS effectively improves sleep. However, the results should not be overestimated. Despite the superior outcome of surgical treatment over conservative treatment for LSS in terms of global PSQI score, we observed no significant difference between the two groups in terms of actual sleep duration throughout the follow-up (Table 4). Moreover, although surgical treatment eliminates the inconvenience of LSS patients due to positional intolerance while sleeping in, nocturnal leg cramps that disturb their sleep and wake them persisted even after surgical treatment. A study which evaluated the surgical outcomes of patients with LSS and nocturnal leg cramps reported that leg cramp improved after surgery in only 18.2% of patients^34^. Multiple factors cause sleep disturbance in patients with LSS, and sleep disturbance observed in our cohort might be associated with depression or other lifestyle factors in addition to chronic pain^35^. The favorable results noticed in such patients cannot be attributed merely to surgical treatment of LSS.

There were no differences in the use of prescribed medication at initial enrollment, whereas analgesics and anticonvulsants were more frequently prescribed in the conservative group after enrollment. Chronic use of such drugs could aggravate sleep disturbance in conservative group. Finally, even after propensity score matching, some independent variables such as dural sac cross-sectional area (mm^2^) showed differences between the two groups even though they were not statistically significant. However, we additionally performed multivariate adjustment including radiologic variables (model 2 in Table 5) after propensity score matching and surgical treatment still showed superior outcome for sleep disturbance despite severe degree of stenosis.

In conclusion, in patients with LSS, sleep quality measured by PSQI was initially improved after treatment, regardless of the treatment method. Sleep quality in the surgical group was remarkably improved early at 6 weeks after surgery and consistently improved during the follow-up period, despite less use of pain killer. In contrast, in the conservative group, improvement of sleep quality at 6-weeks after treatment was not maintained during the follow-up, although the treatment outcome for LSS measured by ODI was continuously improved. Sleep improvement was closely correlated with symptom improvement, especially improvement of leg pain, and multivariate analysis within propensity score matched cohorts identified surgical treatment for LSS patients with sleep disturbance had a significantly greater chance of improving sleep quality than conservative treatment. Subgroup analysis of conservative cohort identified that failure of sleep improvement in conservative group was significantly associated with depression presented by worse score in Hamilton depression rating scale, and more severe degree of foraminal-type stenosis, which should be carefully considered for conservative treatment of LSS patients with sleep disturbance.

## References

[1] Hughes CM (2009). Acupuncture and reflexology for insomnia: A feasibility study. Acupunct. Med..

[2] Kalichman L (2009). Spinal stenosis prevalence and association with symptoms: The Framingham Study. Spine J..

[3] De Villiers P, Booysen E (1976). Fibrous spinal stenosis. A report on 850 myelograms with a water-soluble contrast medium. Clin. Orthop. Relat. Res..

[4] Roberson GH, Llewellyn HJ, Taveras JM (1973). The narrow lumbar spinal canal syndrome. Radiology.

[5] Fanuele JC, Birkmeyer NJ, Abdu WA, Tosteson TD, Weinstein JN (2000). The impact of spinal problems on the health status of patients: Have we underestimated the effect?. Spine.

[6] Lurie, J. & Tomkins-Lane, C. Management of lumbar spinal stenosis. *BMJ* **352**, h6234 (2016). 10.1136/bmj.h6234 PMC6887476 26727925

[7] Binder, D. K., Schmidt, M. H. & Weinstein, P. R. Lumbar spinal stenosis. In *Seminars in Neurology*, Vol. 22 157–166 (Thieme Medical Publishers, Inc., New York, 2002). 10.1055/s-2002-36539 12524561

[8] Cirelli C, Tononi G (2008). Is sleep essential?. PLoS Biol..

[9] Dattilo M (2011). Sleep and muscle recovery: Endocrinological and molecular basis for a new and promising hypothesis. Med. Hypotheses.

[10] Jaussent I (2011). Insomnia and daytime sleepiness are risk factors for depressive symptoms in the elderly. Sleep.

[11] Luyster FS, Strollo PJ, Zee PC, Walsh JK (2012). Sleep: A health imperative. Sleep.

[12] Cappuccio FP, D'Elia L, Strazzullo P, Miller MA (2010). Sleep duration and all-cause mortality: A systematic review and meta-analysis of prospective studies. Sleep.

[13] Schubert CR (2002). Prevalence of sleep problems and quality of life in an older population. Sleep.

[14] Mystakidou K (2007). The relationship of subjective sleep quality, pain, and quality of life in advanced cancer patients. Sleep.

[15] Iliescu EA (2003). Quality of sleep and health-related quality of life in haemodialysis patients. Nephrol. Dial. Transplant..

[16] Broström A, Strömberg A, Dahlström U, Fridlund B (2004). Sleep difficulties, daytime sleepiness, and health-related quality of life in patients with chronic heart failure. J. Cardiovasc. Nurs..

[17] Nunes DM (2009). Impaired sleep reduces quality of life in chronic obstructive pulmonary disease. Lung.

[18] Sezgin M (2015). Sleep quality in patients with chronic low back pain: A cross-sectional study assesing its relations with pain, functional status and quality of life. J. Back Musculoskelet. Rehabil..

[19] Fairbank JC, Pynsent PB (2000). The Oswestry Disability Index. Spine.

[20] Reimer MA, Flemons WW (2003). Quality of life in sleep disorders. Sleep Med. Rev..

[21] Wilcox S (2000). Factors related to sleep disturbance in older adults experiencing knee pain or knee pain with radiographic evidence of knee osteoarthritis. J. Am. Geriatr. Soc..

[22] Khazzam MS, Mulligan EP, Brunette-Christiansen M, Shirley Z (2018). Sleep quality in patients with rotator Cuff disease. J. Am. Acad. Orthop. Surg..

[23] Austin L (2015). Sleep disturbance associated with rotator cuff tear: Correction with arthroscopic rotator cuff repair. Am. J. Sports Med..

[24] Chen AF (2016). Prospective evaluation of sleep disturbances after total knee arthroplasty. J. Arthroplasty.

[25] Zhong W (2015). In vivo dynamic changes of dimensions in the lumbar intervertebral foramen. Spine J..

[26] Kim, J. *et al.* Prevalence of sleep disturbance in patients with lumbar spinal stenosis and analysis of the risk factors. *Spine J.* **20**, 1239–1247 (2020). 10.1016/j.spinee.2020.02.008 32061837

[27] Buysse DJ, Reynolds CF, Monk TH, Berman SR, Kupfer DJ (1989). The Pittsburgh Sleep Quality Index: A new instrument for psychiatric practice and research. Psychiatry Res..

[28] Sohn SI, Kim DH, Lee MY, Cho YW (2012). The reliability and validity of the Korean version of the Pittsburgh Sleep Quality Index. Sleep Breath. Schlaf Atmung.

[29] Cao XL (2017). The prevalence of insomnia in the general population in China: A meta-analysis. PLoS ONE.

[30] Backhaus J, Junghanns K, Broocks A, Riemann D, Hohagen F (2002). Test–retest reliability and validity of the Pittsburgh Sleep Quality Index in primary insomnia. J. Psychosom. Res..

[31] Schizas C (2010). Qualitative grading of severity of lumbar spinal stenosis based on the morphology of the dural sac on magnetic resonance images. Spine.

[32] Lee S (2010). A practical MRI grading system for lumbar foraminal stenosis. AJR Am. J. Roentgenol..

[33] von Elm E (2007). The Strengthening the Reporting of Observational Studies in Epidemiology (STROBE) statement: Guidelines for reporting observational studies. Lancet (Lond. Engl.).

[34] Matsumoto M (2009). Nocturnal leg cramps: A common complaint in patients with lumbar spinal canal stenosis. Spine.

[35] Finan PH, Smith MT (2013). The comorbidity of insomnia, chronic pain, and depression: Dopamine as a putative mechanism. Sleep Med. Rev..

